# Homozygous *SMAD6* variants in two unrelated patients with craniosynostosis and radioulnar synostosis

**DOI:** 10.1136/jmg-2023-109151

**Published:** 2024-01-30

**Authors:** Ilse Luyckx, Isaac Scott Walton, Nele Boeckx, Kristof Van Schil, Chingyiu Pang, Mania De Praeter, Helen Lord, Christopher Mark Watson, David T Bonthron, Lut Van Laer, Andrew O M Wilkie, Bart Loeys

**Affiliations:** 1 Center of Medical Genetics, Faculty of Medicine and Health Sciences, University of Antwerp and Antwerp University Hospital, Antwerp, Belgium; 2 Department of Clinical Genetics, Radboud University Medical Center, Nijmegen, Netherlands; 3 MRC Weatherall Institute of Molecular Medicine, University of Oxford, John Radcliffe Hospital, Oxford, UK; 4 Department of Paediatric Neurosurgery, University Hospital Antwerp, Antwerp, Belgium; 5 Oxford Medical Genetics Laboratories, Oxford University Hospitals NHS Foundation Trust, Churchill Hospital, Oxford, UK; 6 Leeds Institute of Medical Research, University of Leeds, St. James’s University Hospital, Leeds, UK

**Keywords:** Human Genetics, Mutation, Congenital, Hereditary, and Neonatal Diseases and Abnormalities

## Abstract

**Background:**

*SMAD6* encodes an intracellular inhibitor of the bone morphogenetic protein (BMP) signalling pathway. Until now, rare heterozygous loss-of-function variants in *SMAD6* were demonstrated to increase the risk of disparate clinical disorders including cardiovascular disease, craniosynostosis and radioulnar synostosis. Only two unrelated patients harbouring biallelic *SMAD6* variants presenting a complex cardiovascular phenotype and facial dysmorphism have been described.

**Cases:**

Here, we present the first two patients with craniosynostosis harbouring homozygous *SMAD6* variants. The male probands, both born to healthy consanguineous parents, were diagnosed with metopic synostosis and bilateral or unilateral radioulnar synostosis. Additionally, one proband had global developmental delay. Echocardiographic evaluation did not reveal cardiac or outflow tract abnormalities.

**Molecular analyses:**

The novel missense (c.[584T>G];[584T>G], p.[(Val195Gly)];[(Val195Gly)]) and missense/splice-site variant (c.[817G>A];[817G>A], r.[(817g>a,817delins[a;817+2_817+228])];[(817g>a,817delins[a;817+2_817+228])], p.[(Glu273Lys,Glu273Serfs*72)];[(Glu273Lys,Glu273Serfs*72)]) both locate in the functional MH1 domain of the protein and have not been reported in gnomAD database. Functional analyses of the variants showed reduced inhibition of BMP signalling or abnormal splicing, respectively, consistent with a hypomorphic mechanism of action.

**Conclusion:**

Our data expand the spectrum of variants and phenotypic spectrum associated with homozygous variants of *SMAD6* to include craniosynostosis.

## Introduction

Over the past 10 years, *SMAD6* haploinsufficiency has been reported to be significantly associated with discrete human congenital disorders, that is, cardiovascular diseases encompassing conotruncal and left ventricular outflow defects such as bicuspid aortic valve-related aortopathy, craniosynostosis and radioulnar synostosis. The spectrum of associated variants includes rare heterozygous truncating and missense variants located in the functional domains of the protein, determining their disease-causative effect through loss-of-function. However, no genotype-phenotype correlation has emerged, and even identical nucleotide changes have been found in patients with either cardiovascular disease, craniosynostosis or radioulnar synostosis.^1–11^

Craniosynostosis is a developmental craniofacial anomaly characterised by the premature fusion of one or more cranial sutures of the skull. This fusion restricts normal growth of the skull and brain, and, as such, surgical correction is often needed to prevent complications affecting sensory, respiratory and neurological function.^12^ Craniosynostosis occurs in about 1 in 2200 children,^13^ and, approximately one-quarter of patients with craniosynostosis has a molecular diagnosis, with the highest genetic load in syndromic and multisuture cases.^12 14^ Syndromic and non-syndromic patients with a rare heterozygous pathogenic *SMAD6* variant have been described with single and multiple fusions of most major sutures, with metopic synostosis as the most common presentation.^1 8 9^ Remarkably, many *SMAD6*-variant positive individuals remain asymptomatic. A two-locus inheritance model has been proposed to explain reduced penetrance, that is, the co-occurrence of a rare heterozygous pathogenic *SMAD6* variant with a common variant near *BMP2* (rs1884302).^8 9^ However, this hypothesis has not been independently confirmed.^1^ Furthermore, some evidence for an association between *SMAD6* variants and patients combining craniosynostosis with intellectual disability has been described.^15^ So far, one study identified *SMAD6* as a candidate gene for intellectual disability using a meta-analysis of 2104 trios.^16^ Another recent study showed enrichment of *SMAD6* variants in a cohort of autism spectrum disorder/developmental delay patients.^17^ Though, no details on the *SMAD6* variants and associated cranial or neurodevelopmental phenotypes of these patients were provided.^16 17^

Congenital radioulnar synostosis is a rare condition with approximately 500 cases described in the literature.^6 10 18^ This skeletal malformation is characterised by an abnormal connection between the radius and ulna at birth.^18^ Most *SMAD6*-positive carriers with radioulnar synostosis are characterised by bilateral radioulnar synostosis, but unilateral radioulnar synostosis is also observed.^6 10^ The quality of the patient’s life can be improved by corrective surgery and/or medication to control pain.^10^

Finally, *SMAD6*-related cardiovascular disease encompasses a range of cardiac and outflow tract abnormalities including left ventricular obstruction defects, conotruncal defects and a bicuspid aortic valve, the latter of which is associated with late-onset vascular complications such as thoracic aortic aneurysm and dissections.^2–5 7 11^ Advanced surgery to correct the malformation, prophylactic medication and disease monitoring are imperative for these patients.^19–21^ Rare heterozygous pathogenic *SMAD6* variants were shown to be enriched in patients with left ventricular obstruction defects.^2 4 7 11^

In 2019, a case report^3^ described two unrelated patients with biallelic missense variants in *SMAD6*, who both presented with a complex cardiac phenotype and facial dysmorphism. Cardiovascular abnormalities included aortic isthmus stenosis in the male patient (born to consanguineous parents) with a homozygous *SMAD6* variant (p.[(Ile466Thr)];[(Ile466Thr)]), while the female proband, harbouring compound heterozygous *SMAD6* variants (p.[(Phe357Ile)];[(Ser483Pro)]), had a dysplastic and stenotic pulmonary valve, dilated cardiomyopathy, narrowing of the proximal left pulmonary artery, stenosis of the left main coronary artery leading to ischaemia and a venous anomaly in the brain. Facial dysmorphism was described in both probands. Additionally, the male patient presented with bilateral radioulnar synostosis, syndactyly, unilateral renal hypoplasia and global developmental delay. Interestingly, craniosynostosis was not observed in either proband or any of their parents. Careful examination did reveal some mild signs of cranial deformity in both probands, that is, microcephaly with a flattened forehead or bitemporal narrowing with triangular shape of the forehead.

**Figure 1:**
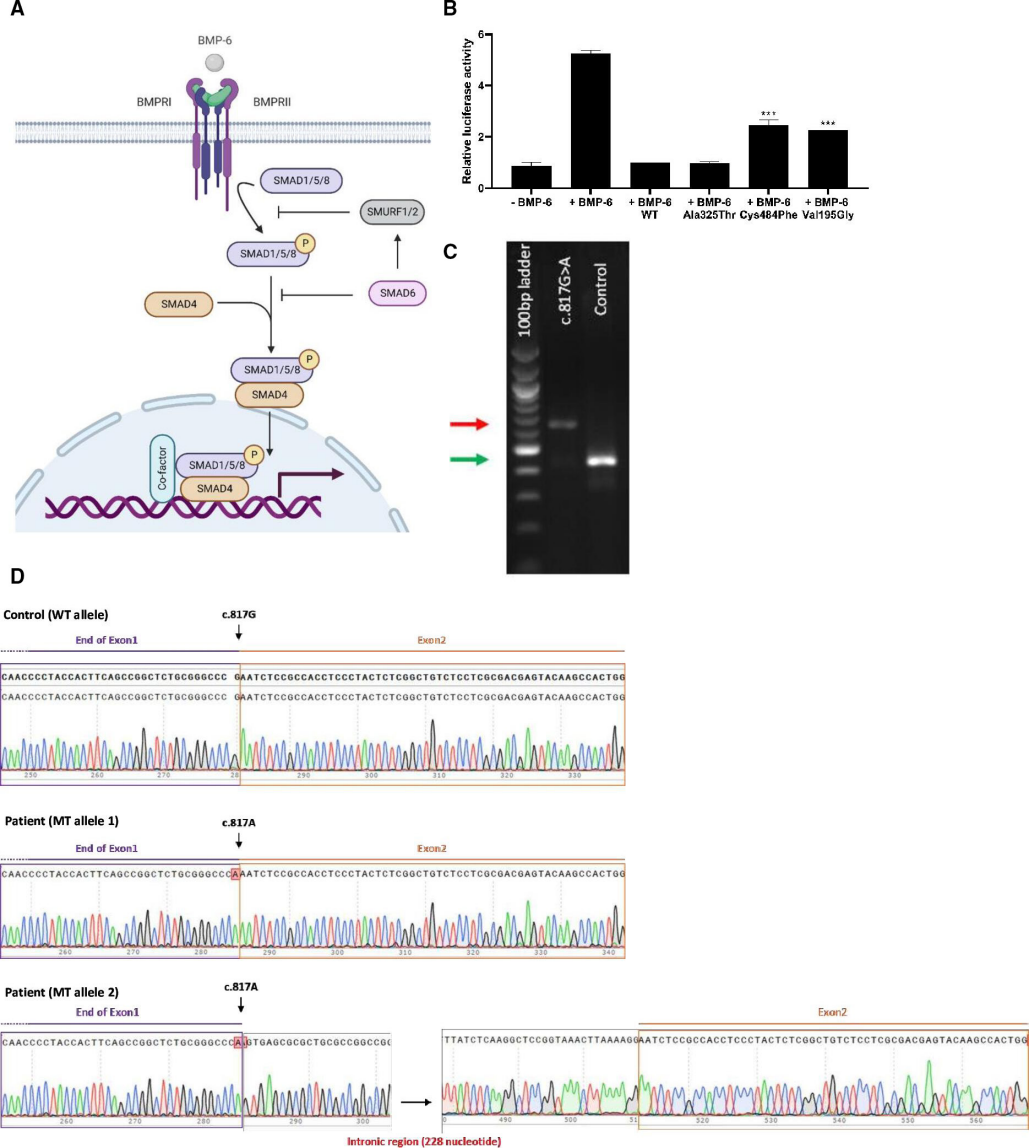
Functional analyses of novel *SMAD6* variants. (A) Schematic overview of the SMAD-dependent bone morphogenetic protein (BMP) signalling pathway (simplification). On BMP ligand binding, specific type I and type II receptors form a heterotetrameric complex. The type II receptor phosphorylates the type I receptor, which, in turn, phosphorylates SMAD1, SMAD5 and SMAD8 (ie, canonical BMP signalling). Phosphorylated Smads propagate the signal via complex formation with Smad4 and translocate into the nucleus, where the expression of BMP-responsive target genes is regulated by binding of the complex to DNA. Canonical BMP signalling is intracellularly inhibited by inhibitory Smads (SMAD6, SMAD7) and E3 ubiquitin ligases like Smurf1 and Smurf2. Created with BioRender.com, and the figure was exported under a paid subscription. (B) Inhibitory effect of *SMAD6* variants on BMP signalling measured via the BRE-luc transcriptional reporter. On BMP-6 ligand stimulation (12 hours), firefly luciferase activity of the BRE-luc reporter was measured in C2C12 cells. Graphs represent means±SEM from three independent experiments. The SMAD6 constructs were either wild type (WT) or contained one of the following variants: p.(Ala325Thr) (negative control), p.(Cys484Phe) (positive control) or p.(Val195Gly) (novel identified variant). Data were normalised using *Renilla* levels, relativised to WT SMAD6, and analysed by one-way analysis of variance with Dunnett’s multiple comparison test (***p value ≤0.001) (GraphPad Prism V.9.3.1). (C) Amplification of cDNA from the lymphoblastoid cell line of Proband 2 (c.[817G>A];[817G>A]) demonstrates a normal (indicated with green arrow, barely visible) and abnormal *SMAD6* splice product (indicated with red arrow). The abnormal splice product was absent in a control sample. (D) Dideoxy-sequencing of amplified cDNA corresponding to normal splice product in control sample (top panel), normal splice product in c.817G>A sample (middle panel), and abnormal splice product in c.817G>A sample (bottom panel). This confirmed a sole WT (c.817G) product in the control sample, a mutant (c.817G>A) product with similar size as WT product in the patient sample, and a larger mutant product (corresponding to 228 nucleotide retention of the 5′ end of the intron) in the patient sample.

The gene *SMAD6* encodes an important intracellular inhibitor of the bone morphogenetic protein (BMP) signalling pathway (figure 1A).^22^ To activate the pathway, BMP ligands, for example, BMP-6, bind to serine-threonine kinase receptors, known as type I and type II receptors, which form heterotetrameric signalling complexes with two receptors of each subtype. On complex formation, the type II receptors transphosphorylate specific residues of the type I receptor, causing type I receptor activation and phosphorylation of receptor-regulated SMAD effector proteins, such as SMAD1, SMAD5 and SMAD8 (ie, canonical signalling).^23^ Subsequently, phosphorylated Smads form heteromeric complexes with a common mediator (SMAD4) and translocate into the nucleus, where the complex regulates target gene expression via binding to DNA-binding SMAD elements such as the BMP/SMAD responsive element (BRE). The translocated heteromeric complex might also indirectly regulate gene expression by, for example, interacting with DNA-binding transcription factors.^24^ SMAD6 controls canonical BMP signalling by inducing degradation of receptors and regulatory-Smads, together with E3 ubiquitin ligases such as SMURF1 and SMURF2.^25^

In this study, we report two male patients with metopic synostosis and radioulnar synostosis harbouring homozygous variants in *SMAD6* identified by either trio-based whole-exome sequencing (WES) or targeted sequencing. Additionally, one proband presented with global developmental delay. Neither proband had cardiac or outflow tract defects, as assessed using echocardiography. Functional analysis of the MH1-domain locating missense and putative splice-site variant revealed a pathogenic effect on BMP signalling activity and protein stability, or splicing, respectively.

## Methods

The detailed methods for molecular analyses are described in online supplemental information. In brief, trio-based WES or targeted *SMAD6* Sanger sequencing was performed to identify the pathogenic variant. Sanger sequencing was performed for validation and/or segregation analysis. Functional assessment of the novel variants included an improved transcriptional assay and a protein stability assay (proband 1) and examination of aberrant splicing in the patient’s lymphoblastoid cell line (proband 2).

10.1136/jmg-2023-109151.supp1 Supplementary data

## Results

### Proband 1

Proband 1 is the first child of healthy consanguineous parents of Moroccan origin. Clinical data are summarised in table 1 and images of the proband are depicted in online supplemental figure S1. At birth, he was diagnosed with premature closure of the metopic suture leading to trigonocephaly, for which he underwent surgery (fronto-orbital advancement and remodelling) at the age of 9 months. Physical examination also revealed limitation of pronation and supination of the left elbow. Plain radiographs confirmed left-sided radioulnar synostosis. His medical history is also significant for cryptorchidism with circumcision surgery at 13 months and he was diagnosed with factor XI deficiency. No evidence of raised intracranial pressure was found on ophthalmological examination, but he had right eye astigmatism for which glasses were prescribed. Echocardiographic evaluation up to 40 months of age did not reveal outflow tract or cardiac anomalies, but was suggestive of a left upper vena pulmonalis ending in the vena innominate (ie, abnormal pulmonary vein morphology). Up to 38 months of age, his anthropometric parameters evolved within normal limits but developmental delay was noted. Formal testing at 38 months revealed a total developmental quotient of 70 (second percentile) with significant language development delay (age-equivalent level 18 months) and disharmonic motor development (fine motor 36 months, gross motor 25 months).

**Table 1:** Clinical and genetic characteristics of probands with biallelic variants in *SMAD6*

	This study Proband 1	This study Proband 2	Kloth *et al*, 2019 Proband 1	Kloth *et al*, 2019 Proband 2
Genotype	Homozygous	Homozygous	Homozygous	Compound heterozygous
Chromosomal position (hg38)	chr15:66703842T>G; chr15:66703842T>G	chr15: 66 704 075G>A; chr15: 66 704 075G>A	chr15: 67073779T>C; chr15: 67073779T>C	chr15: 67073451T>A; chr15: 67073829T>C
Nucleotide change	c.584T>G; c.584T>G	c.817G>A; c.817G>A	c.1397T>C; c.1397T>C	c.1069T>A; c.1447T>C
Protein change	p.(Val195Gly); p.(Val195Gly)	p.[(Glu273Lys,Glu273Serfs*72)];[(Glu273Lys,Glu273Serfs*72)])	p.(Ile466Thr); p.(Ile466Thr)	p.(Phe357Ile); p.(Ser483Pro)
Type	Missense	Missense/splice-site	Missense	Missense; missense
Protein domain	MH1	MH1	MH2	MH2; MH2
CADD score (v1.6)	31	35	28.8	31; 28.9
gnomAD (v2.1.1)	Absent	Absent	Absent	Absent
Family history	Healthy parents	Healthy parents	Healthy parents	Healthy parents
Consanguinity	Yes	Yes	Yes	No
Sex	Male	Male	Male	Female
Age at last follow-up	Early childhood	Early childhood	Middle childhood	Middle childhood
Craniosynostosis	Metopic synostosis	Metopic synostosis	Absent	Absent
Global developmental delay	Yes	No	Yes^†^	No
Gross motor delay	Yes	No	Slightly delayed^‡^	No
Speech delay	Yes	No	Slightly impaired^§^	No
Cardiac and outflow tract abnormalities	No	No	Coarctation of the aorta	Dysplastic pulmonary valve, pulmonic stenosis, coronary artery stenosis (ie, left main coronary artery), pulmonary artery stenosis, dilated cardiomyopathy
Radioulnar synostosis	Left-sided	Bilateral	Bilateral	Absent
Other	Cryptorchidism, abnormal pulmonary vein morphology, factor XI deficiency, astigmatism	None	Facial dysmorphism (eg, microcephaly, abnormality of the ears, etc), unilateral renal hypoplasia, toe syndactyly (bilateral), dorsal hirsutism, EEG¶ abnormality	Facial dysmorphism (eg, low anterior hairline, prominent nose, short neck, abnormality of the ears, etc), transient neutropenia, decreased circulating antibody level

Reference build, GRCh38; RefSeq NM_005585.5.

Human Phenotype Ontology (HPO) terms have been used to annotate the phenotypes mentioned in the table.

*Also associated with abnormal (p.(Glu273Serfs*72)) splicing, see text and figure 1C,D.

†With intellectual disability.

‡Sitting at 8 months, crawling at 10 months, walking at 19 months.

§Speaks 3–4 word sentences, uses fantasy language.

¶EEG, electroencephalography.

In proband 1, we identified a homozygous *SMAD6* variant using trio-based WES (table 1 (online supplemental table 1 and 2). This variant (c.[584T>G];[584T>G], p.[(Val195Gly)];[(Val195Gly)]) has not been reported in healthy individuals (gnomAD v2.1.1) and has a CADD score of 31. Both parents were confirmed to be heterozygous for the variant. To assess the inhibitory activity of mutant SMAD6, a dual-luciferase assay,^7^ able to discriminate damaging from non-damaging variants, was performed in C2C12 cells using a transcriptional reporter construct containing a BMP-responsive element (figure 1B).^25 26^ Previously reported controls, that is, positive (p.(Cys484Phe)) and negative (p.(Ala325Thr)),^1 7^ along with the SMAD6 mutant (p.(Val195Gly)) construct, were evaluated on specific BMP pathway stimulation with BMP-6 ligand (R&D Systems). This is different from the previous assay, in which a constitutively active mutant version of the BMPR1A receptor was used. The p.(Val195Gly) missense variant showed reduced inhibitory capacity in an established transcriptional reporter assay containing a BMP-responsive element (p value ≤0.001), similar to the positive control. More precisely, a 50% reduction in BMP signalling activity was observed for the p.(Val195Gly) SMAD6 mutant, as compared with the WT. The negative control maintained potent inhibitory activity similar to WT. Additionally, the stability of the mutant SMAD6 protein was tested on a immunoblot using aliquots of cell lysates produced in the BRE-luc transcriptional reporter assay (online supplemental figure S2). The p.(Val195Gly) missense variant showed significantly reduced SMAD6 protein levels (p value <0.0001), indicative of protein instability.

### Proband 2

Proband 2 was the second child of healthy consanguineous parents of Pakistani origin. His parents observed that his forehead was pointed from the time of birth and also noted that his elbows did not fully straighten. When assessed at 6 months of age, he presented with a trigonocephalic head shape and unusual creases around the elbows. Radiological imaging showed the presence of metopic synostosis and bilateral proximal radioulnar synostosis with dislocation of the left radial head. The metopic synostosis was confirmed by CT scan at age 25 months and no other cranial abnormalities were noted. His parents declined surgical treatment of the trigonocephaly. A formal developmental assessment at age 18 months indicated abilities within the normal range. Echocardiography at age 21 months showed a normal heart and outflow tract. His last examination at 3 years did not reveal new or progressing symptoms.

In proband 2, we identified a homozygous variant (c.[817G>A];[817G>A] predicting p.[(Glu273Lys)]; [(Glu273Lys)]) using targeted *SMAD6* sequencing (table 1 (online supplemental table 1 and 2). Both parents were confirmed to be heterozygous for the variant. This variant, which locates at the last nucleotide of exon 1, has not been reported in healthy individuals (gnomAD v2.1.1), has a CADD score of 35, and splice prediction tool Alamut (Alamut Visual Plus v.1.4) predicts abnormal splicing due to failure of proper recognition of intron 1 splice donor (online supplemental figure S3). A different nucleotide substitution at the same position (c.817G>C), identified in heterozygous state in a child with syndromic sagittal synostosis, was previously shown to disrupt normal splicing between exons 1 and 2 in the publication of Calpena *et al*.^1^ Analysis of cDNA derived from messenger RNA using primers located in exons 1 and 3 confirmed abnormal splicing, as a larger abnormal product was detected in patient’s lymphoblastoid cell line, and not in a healthy control cells (figure 1C). Subsequent dideoxy-sequencing of PCR products showed the presence of trace amounts of the *SMAD6* transcript harbouring the variant (c.817G>A), with partial intron retention in the amplicon containing the abnormal splice product due to transcript read-through of 228 nucleotides into intron 1 (figure 1D). The formal nomenclature of the major RNA product is r.[(817g>a,817delins[a;817+2_817+228])];[(817g>a,817delins[a;817+2_817+228])] encoding a frameshifted protein p.[(Glu273Serfs*72)];[(Glu273Serfs*72)]. Importantly, the residual normally spliced product would be predicted to encode a missense change, p.(Glu273Lys).

## Discussion

In this report, we describe two patients, both harbouring a homozygous damaging *SMAD6* variant, and both affected with metopic craniosynostosis and radioulnar synostosis, but without typical cardiac and outflow tract abnormalities. As noted in the Introduction section, biallelic *SMAD6* variants were previously described in two patients, both of whom displayed a complex cardiovascular phenotype comprising abnormalities in the cardiac valves and aorta, coronary vessels and a venous anomaly in the brain.^3^ At present, it is difficult to assess the contribution of SMAD6 deficiency to all cardiovascular-related pathologies due to lack of data. Combining the data in this report, with the available phenotypic data of *SMAD6*-variant positive patients, it remains questionable that a venous anomaly in the brain (literature) or inflow tract abnormality (proband 1) are part of the *SMAD6* variant spectrum, for which further research is warranted. Craniosynostosis was present in both patients described here, unlike those previously reported with biallelic *SMAD6* variants. Our data support the current observation that there is no increased prevalence of thoracic aortic aneurysmal disease in *SMAD6*-variant positive families with craniosynostosis or vice versa. Additionally, the parents of both probands in this study, who did not present with clinical features, harbour the respective *SMAD6* variant in heterozygous state.

A subset of the previously reported patients with SMAD6-associated radioulnar synostosis have been described with abnormal head shapes too, including frontal bossing.^6^ Our two cases with craniosynostosis also presented with radioulnar synostosis, suggesting that a clinical overlap is emerging. The additional finding of coagulation abnormality in the male proband homozygous for the p.(Val195Gly) variant might be explained by homozygosity at other loci due to consanguinity. For example, our patient was found also to carry a homozygous rare missense variant in the *F11* gene (c.[1603G>C];[1603G>C], p.[(Ala535Pro);(Ala535Pro)]) (absent from gnomAD), possibly explaining the factor XI deficiency. Similarly, homozygosity at other loci might explain global developmental delay in this patient. Alternatively, this observation could also support an association of SMAD6 deficiency with neurodevelopmental disease, as already suggested in the literature.^16^

Functional assessment of variants is key in answering puzzling questions regarding the variability in clinical expressivity and unpredictable penetrance. Despite protein instability, we demonstrated that the p.(Val195Gly) variant, located in the MH1-domain, did not completely abolish its inhibitory effect on BMP signalling, as some residual inhibitory effect is still apparent. Previously, only MH2 domain locating missense variants exerting a damaging effect on BMP signalling activity could be assayed using a constitutively active mutant receptor in a transcriptional assay. With our assay, we specifically rely on ligand-dependent binding to wild-type (WT) receptors allowing to model more *SMAD6* variants, and as such, have a more generalisable assay with advantages for variant modelling. The splice-site variant (c.817G>A) generates an abnormal splice product and a normally spliced product, which is predicted to encode a SMAD6 protein encoding p.(Glu273Lys). The latter product might exert some residual inhibitory function too. Supportive evidence for why residual WT activity is needed for viability is found in the publication of Galvin *et al*,^27^ in which a subset of homozygous mice lacking *Smad6* expression die before the age of weaning. Surviving animals developed multiple cardiovascular abnormalities including hyperplasia of the cardiac valves and outflow tract septation defects. No craniosynostosis or radioulnar synostosis were observed in this mouse model; however, it is possible that these phenotypes could have been overlooked.

## Conclusion

To conclude, our report expands the spectrum of phenotypes identified in individuals with homozygous *SMAD6* variants to include craniosynostosis, and emphasises the importance of complementing genetic studies with functional assessment for variant interpretation. In-depth follow-up work will be essential to delineate the overall contribution of *SMAD6* variants to the pathogenesis and to understand the causal mechanisms underlying the extreme clinical variability.
