# Supplementary material for: Homozygous SMAD6 variants in two unrelated patients with craniosynostosis and radioulnar synostosis
Source: J Med Genet. 2024 Jan 30;61(4):363–8. doi: 10.1136/jmg-2023-109151 (PMC10982635; doi:10.1136/jmg-2023-109151)
Supplement: Supplementary data [file jmg-2023-109151supp001.pdf]

Supplementary Information

Homozygous *SMAD6* variants in two unrelated patients with craniosynostosis and radioulnar synostosis

Ilse Luyckx[1,2], Isaac S. Walton[3], Nele Boeckx[1], Kristof Van Schil[1], Chingyiu Pang[3], Mania De Praeter[4], Helen Lord[5], Christopher M. Watson[6], David T. Bonthron[6], Lut Van Laer[1], Andrew O.M. Wilkie[3] and Bart L. Loeys[1,2]

[1]Centre of Medical Genetics, Faculty of Medicine and Health Sciences, University of Antwerp and Antwerp University Hospital, Antwerp, Belgium; [2]Department of Clinical Genetics, Radboud University Medical Center, Nijmegen, The Netherlands; [3]MRC Weatherall Institute of Molecular Medicine, University of Oxford, John Radcliffe Hospital, Oxford, United Kingdom; [4]Department of Paediatric Neurosurgery, University Hospital Antwerp, Antwerp, Belgium; [5]Oxford Medical Genetics Laboratories, Oxford University Hospitals NHS Foundation Trust, Churchill Hospital, Oxford, United Kingdom; [6]Leeds Institute of Medical Research, University of Leeds, St. James's University Hospital, Leeds, United Kingdom

Supplementary Material and Methods: Detailed methods for molecular analyses

Supplementary Table S1 | Classification of the novel identified SMAD6 variants in this study using guidelines of American College of Medical Genetics

Supplementary Table S2 | Primer sequences to generate constructs

Supplementary Figure S1 | Clinical images of Proband 1

Supplementary Figure S2 | Analysis of SMAD6 protein stability

Supplementary Figure S3 | Predicted cryptic splice donor sites downstream *SMAD6* exon 1 (Alamut Visual Plus v.1.4)

Supplementary Material and Methods: Detailed methods for molecular analyses

Sequencing, bioinformatics analysis and variant validation

Genomic DNA was isolated from peripheral blood samples. Trio-based whole exome sequencing was performed with Illumina sequencing technology after enrichment of all exons (SureSelect Human All Exon V5 kit) according to the manufacturer's protocol (Agilent Technologies, USA). Raw data were processed using an in-house developed Galaxy-based pipeline, followed by variant calling with the Genome Analysis Toolkit Unified Genotyper [1]. Variants were subsequently annotated and filtered with the in-house developed ANNOVAR-based database VariantDB [2]. Heterozygous coding or splice site variants (recessive/de novo) were selected, and classified using the ACMG guidelines [3]. The variant was validated using dideoxy-sequencing, as described in [4]. Targeted *SMAD6* sequencing, and subsequent data analysis, were performed as previously described in Calpena et al. [5]. Finally, segregation analysis was performed in parents to confirm inheritance.

Constructs, cell culture and transfection

The following plasmids were used for functional evaluation: pCMV5-FLAG-SMAD6 containing the human cDNA open reading frame of SMAD6 with N-terminal FLAG tag (ID 19766, MRC PPU Reagents and Services, University of Dundee, UK); pRL-TK containing Renilla luciferase reporter; pGL3-BRE-luc containing BMP/SMAD responsive elements (kindly provided by Prof. Marie-José Goumans (Leiden University Medical Center, Netherlands); and the empty vector pcDNA3.1. The Q5-Mutatgenesis kit protocol (E0554, New England Biolabs) was used to generate the mutant constructs (i.e. positive/negative control and variant of interest) using primers listed in Table S1 and the pCMV5-FLAG-SMAD6 plasmid as template. All the generated constructs were verified by dideoxy-sequencing.

Cells (patient's lymphoblastoid cell line, C2C12 cells (kindly provided by Prof. Marie-José Goumans)) were cultured in DMEM supplemented with L-glutamine, penicillin-streptomycin and 10% foetal bovine serum at 37°C under 5% CO_2. C2C12 cells were transiently transfected using ViaFECT (E4981, Promega) according to the manufacturer's instructions (ViaFect:DNA (4:1) ratio).

Functional analyses

1. Splicing analysis using reverse transcriptase polymerase chain reaction

The splice-site variant c.817G>A was analysed with the respective proband's lymphoblastoid cell line (Proband 2) and control, as previously described using reverse transcriptase polymerase chain reaction (RT-PCT) [5]. Fragments visible after agarose gel electrophoresis (Figure 1c) were excised, extracted (NEB Monarch DNA Gel Extraction Kit) and dideoxy-sequenced using the forward primer.

2. Investigation of BMP signalling activity using a dual-luciferase assay

To measure SMAD6-mediated inhibition of BMP signalling, dual luciferase assays were performed based on a previously described system [6] using a luciferase transcriptional reporter construct containing BMP/SMAD responsive elements (BRE-luc) [7 8] and utilizing a full-length SMAD6 construct instead of the shorter isoform previously described [6]. Using the C2C12 mouse muscle myoblast cell line, BMPs were shown to activate the BRE-luc reporter in a strictly dose-dependent manner [8]. In the assay of this study, C2C12 cells in a 12-well plate were liposome-mediated transfected 24 hr after seeding with antibiotic-free medium (DMEM + 10% FBS) containing pGL3-BRE-luc (reporter; 200 ng/well) and either wild-type (WT; 250 ng/well) or mutant *SMAD6* constructs (250 ng/well). The pRL-TK vector was co-transfected to normalise for the transfection levels, and an empty vector (pcDNA3.1)

was used to equalise the total amount of plasmids (500 ng) for each well. 24 hr post-transfection, medium was replaced with complete cell culture medium. On the following day, the BMP pathway was specifically stimulated with 150 ng/ml BMP-6 (6325-BM-020/CF, R&D Systems) for 12 hr using starvation medium (i.e. DMEM + 1% FBS + 2% Penicillin-Streptomycin). Optimal concentration of BMP6 was determined based on a dose-response curve. Luciferase activities were measured in lysates using the Dual-Glo Luciferase Reporter Assay System (E2920, Promega). Data were normalized using Renilla luciferase activity and relativised to the WT.

3. Investigation of protein stability using western blot

The stability of SMAD6 protein was measured on a immunoblot. In brief, aliquots of cell lysates produced in the BRE-luc transcriptional reporter assay were used for standard SDS-PAGE (Mini-Protean TGX 4-15% gradient gels, Bio-Rad) and western blotting (Immobilon-P PVDF membranes, Millipore) procedures. The used primary antibodies were anti-FLAG (F1804, Sigma-Aldrich), to target the SMAD6 protein, and anti-GAPDH (3683, Cell Signalling). For quantitation, captured bands were analysed with Image J software (National Institutes of Health, Bethesda, MD) of four biological replicates.

Supplementary Table S1 | Classification of the novel identified *SMAD6* variants in this study using guidelines of American College of Medical Genetics

Variant	Classified Effect	Protein domain	Evidence
p.(Val195Gly)	Likely pathogenic (II)	MH1	PM1, PM2, PS3, PP3
p.(Glu273Lys)	Likely pathogenic (II)	MH1	PM1, PM2, PS3, BP4

Reference build, GRCh38; RefSeq NM_005585.5

https://www.medschool.umaryland.edu/Genetic_Variant_Interpretation_Tool1.html/

Supplementary Table S2 | Primer sequences to generate constructs

Variant	Forward primer (5' – 3')	Reverse (5' – 3')
SMAD6 – p.Ala325Thr<br>Negative control	GTCTCCGGACaCCACCAAGCC	ATGCTGGCGTCTGAGAATTC
SMAD6 – p.Cys484Phe<br>Positive control	ATCACCTCCTtCCCCTGCTGG	GAACTGCCGGGAGTAGCA
SMAD6 – p.Val195Gly<br>Variant of interest	CTGGAGGCGGgGGAGTCCCGC	CAGCGTGTCCAGCGAGCGC

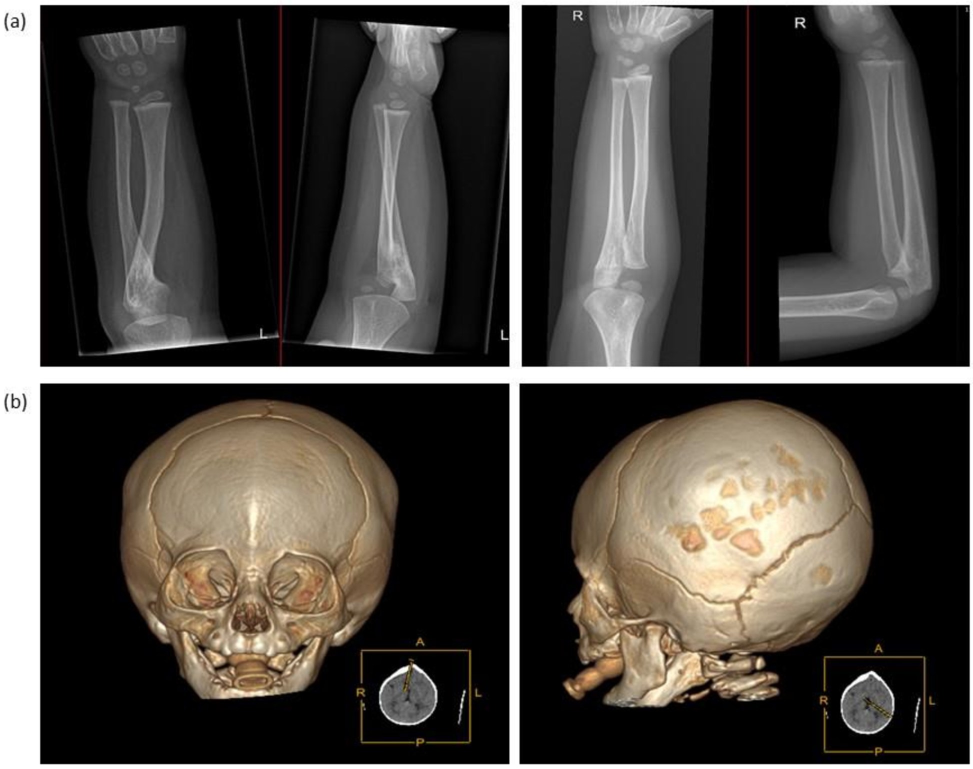

Supplementary Figure S1 | Clinical images of Proband 1

(a) X-ray images of the forearms. Left-sided radioulnar synostosis is confirmed on X-ray images (L: left side (26 months); R: right side (42 months)). (b) Reconstruction of the skull using computed tomography images (6 months). The metopic suture is premature fused leading to trigonocephaly.

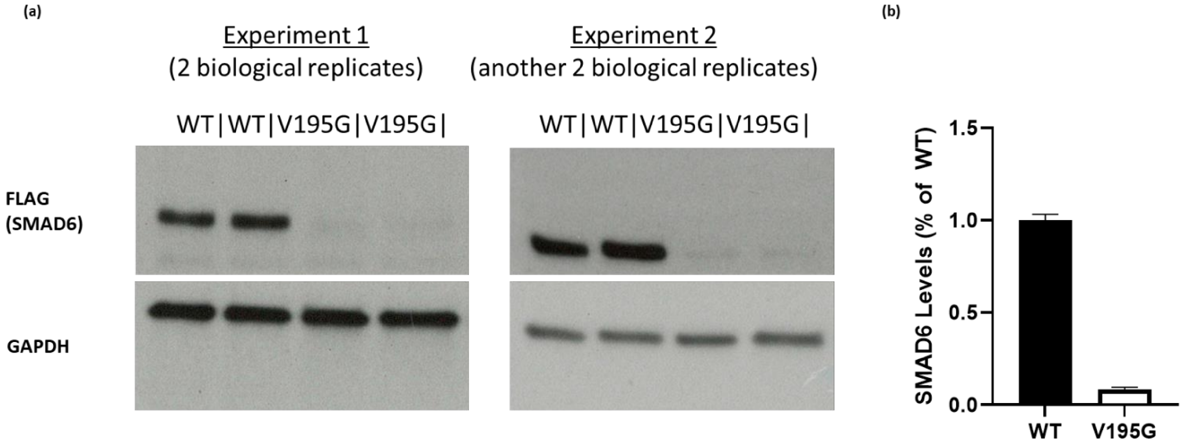

Supplementary Figure S2| Analysis of SMAD6 protein stability

(a) Representative pictures of immunoblots using aliquots of protein extracts from the luciferase assay (two biological replicates). Detected with anti-FLAG antibody, SMAD6 protein levels are compared with

anti-GAPDH loading control. (b) Data were normalised (using GAPDH) and relativised to the WT. The bars represent means ± SEM from four independent experiments ($p<0.0001$, two-tailed unpaired t-test; GraphPad Prism 9.5.1.).

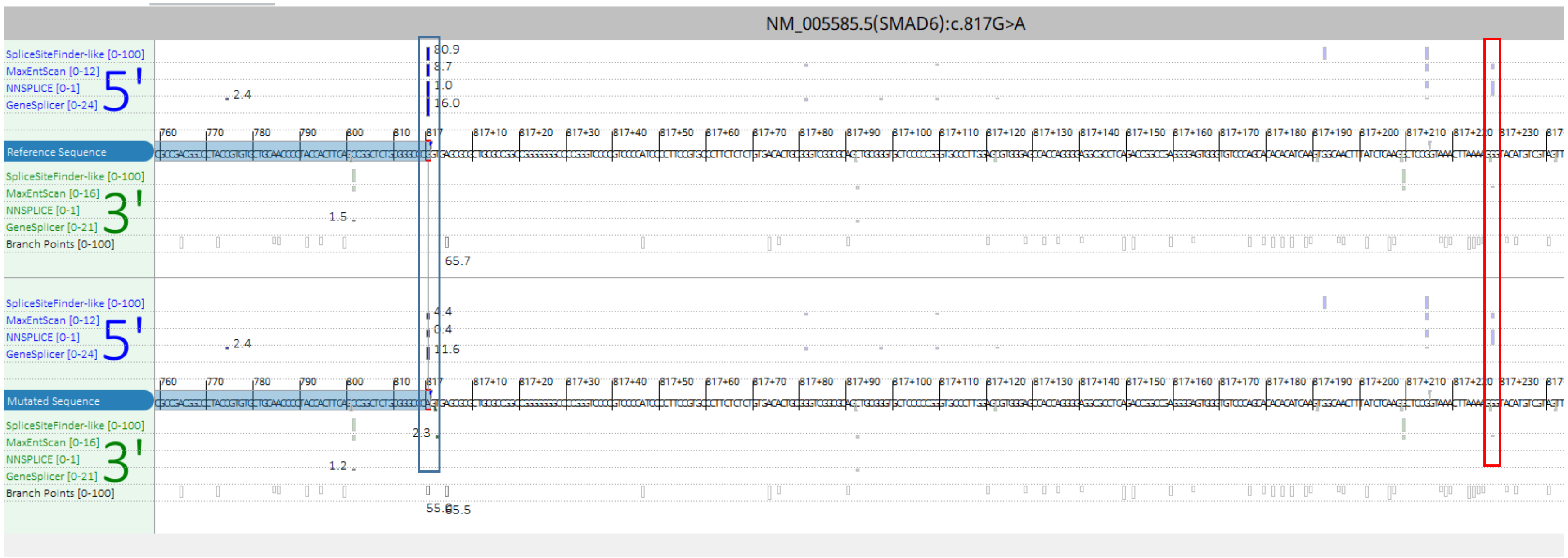

Supplementary Figure S3 | Predicted cryptic splice donor sites downstream *SMAD6* exon 1 (Alamut Visual Plus v.1.4)

Genomic sequence of the 3’ end of *SMAD6* exon 1 and the 5’ part of intron 1 (until position 817+289). The splice site variant (c.817G>C; blue box) is predicted to affect canonical donor splice site by SpliceSiteFinder-like, MaxEntScan, NNSPLICE, and GeneSplicer. Additional cryptic splice donor sites are predicted, including the experimentally validated site at position 228 (red box; NNSPLICE (0.87) and MaxEntScan (3.77)).

References

1. DePristo MA, Banks E, Poplin R, et al. A framework for variation discovery and genotyping using next-generation DNA sequencing data. Nature genetics 2011;**43**(5):491-8
2. Vandeweyer G, Van Laer L, Loeys B, et al. VariantDB: a flexible annotation and filtering portal for next generation sequencing data. Genome medicine 2014;**6**(10):74 doi: 10.1186/s13073-014-0074-6[published Online First: Epub Date]|.
3. Richards S, Aziz N, Bale S, et al. Standards and guidelines for the interpretation of sequence variants: a joint consensus recommendation of the American College of Medical Genetics and Genomics and the Association for Molecular Pathology. Genet Med 2015;**17**(5):405-24 doi: 10.1038/gim.2015.30[published Online First: Epub Date]|.
4. Gillis E, Kumar AA, Luyckx I, et al. Candidate Gene Resequencing in a Large Bicuspid Aortic Valve-Associated Thoracic Aortic Aneurysm Cohort: SMAD6 as an Important Contributor. Frontiers in physiology 2017;**8**:400 doi: 10.3389/fphys.2017.00400[published Online First: Epub Date]|.
5. Calpena E, Cuellar A, Bala K, et al. SMAD6 variants in craniosynostosis: genotype and phenotype evaluation. Genet Med 2020;**22**(9):1498-506 doi: 10.1038/s41436-020-0817-2[published Online First: Epub Date]|.
6. Tan HL, Glen E, Topf A, et al. Nonsynonymous variants in the SMAD6 gene predispose to congenital cardiovascular malformation. Hum Mutat 2012;**33**(4):720-7 doi: 10.1002/humu.22030[published Online First: Epub Date]|.
7. Goto K, Kamiya Y, Imamura T, et al. Selective inhibitory effects of Smad6 on bone morphogenetic protein type I receptors. The Journal of biological chemistry 2007;**282**(28):20603-11 doi: 10.1074/jbc.M702100200[published Online First: Epub Date]|.
8. Korchynskyi O, ten Dijke P. Identification and functional characterization of distinct critically important bone morphogenetic protein-specific response elements in the Id1 promoter. The Journal of biological chemistry 2002;**277**(7):4883-91 doi: 10.1074/jbc.M111023200[published Online First: Epub Date]|.
